# Does Extracellular DNA Production Vary in Staphylococcal Biofilms Isolated From Infected Implants versus Controls?

**DOI:** 10.1007/s11999-017-5266-0

**Published:** 2017-02-13

**Authors:** Beata Zatorska, Marion Groger, Doris Moser, Magda Diab-Elschahawi, Luigi Segagni Lusignani, Elisabeth Presterl

**Affiliations:** 1 Department of Hospital Hygiene and Infection Control, Medical University of Vienna, Vienna, Austria; 2 Department of Core Facilities, Medical University of Vienna, Vienna, Austria; 3 Department of Cranio-Maxillofacial and Oral Surgery, Medical University of Vienna, Vienna, Austria

## Abstract

**Background:**

Prosthetic implant infections caused by *Staphylococcus aureus* and *epidermidis* are major challenges for early diagnosis and treatment owing to biofilm formation on the implant surface. Extracellular DNA (eDNA) is actively excreted from bacterial cells in biofilms, contributing to biofilm stability, and may offer promise in the detection or treatment of such infections.

**Questions/purposes:**

(1) Does DNA structure change during biofilm formation? (2) Are there time-dependent differences in eDNA production during biofilm formation? (3) Is there differential eDNA production between clinical and control Staphylococcal isolates? (4) Is eDNA production correlated to biofilm thickness?

**Methods:**

We investigated eDNA presence during biofilm formation in 60 clinical and 30 control isolates of *S aureus* and *S epidermidis.* The clinical isolates were isolated from patients with infections of orthopaedic prostheses and implants: 30 from infected hip prostheses and 30 from infected knee prostheses. The control isolates were taken from healthy volunteers who had not been exposed to antibiotics and a hospital environment during the previous 3 and 12 months, respectively. Control *S epidermidis* was isolated from the skin of the antecubital fossa, and control *S aureus* was isolated from the nares. For the biofilm experiments the following methods were used to detect eDNA: (1) fluorescent staining with 4′,6-diamidino-2-phenylindole (DAPI), (2) eDNA extraction using a commercial kit, and (3) confocal laser scanning microscopy for 24-hour biofilm observation using propidium iodide and concanavalin-A staining; TOTO^®^-1 and SYTO^®^ 60 staining were used for observation and quantification of eDNA after 6 and 24 hours of biofilm formation. Additionally antibiotic resistance was described.

**Results:**

eDNA production as observed by confocal laser scanning microscopy was greater in clinical isolates than controls (clinical isolates mean ± SD: 1.84% ± 1.31%; control mean ± SD: 1.17% ± 1.37%; p < 0.005) after 6 hours of biofilm formation. After 24 hours, the amount of eDNA was greater in biofilms of *S epidermidis* than in biofilms of *S aureus* (*S aureus* mean ± SD: 1.35% ± 2.0%; *S epidermidis* mean ± SD: 6.42% ± 10.6%; p < 0.05). Clinical isolates of *S aureus* and *S epidermidis* produced more eDNA than control isolates at 6 hours of biofilm formation. The extraction method also showed that clinical isolates produced substantially greater amounts of eDNA than controls.

**Conclusions:**

*S aureus* and *S epidermidis* exhibit a differential production of DNA with time. Clinical isolates associated with implant infections produce greater amounts of eDNA than controls. Future research might focus on the diagnostic value of eDNA as a surrogate laboratory marker for biofilm formation in implant infections.

**Clinical relevance:**

eDNA should be considered as a potential future diagnostic tool or even a possible target to modify biofilms for successful treatment of biofilm-associated infections.

## Introduction

Microbial biofilms are an important factor in the pathophysiology of prosthetic joint infections. Data show that prosthetic joint infections account for 25% of failed knee arthoplasties and 15% of failed hip arthroplasties [1, 2, 5]. Patients may be faced with prolonged and often futile antimicrobial treatment where implant removal is ultimately inevitable [8, 32]. Biofilms are a natural niche in which bacteria may survive in an adverse environment [17]. After initial bacterial adhesion on the implant surface, colonization and proliferation occurs. Producing extracellular matrix consisting of extrapolymeric substances, for example, exopolysaccharides, proteins, and nucleic acids, bacteria exhibit several mechanisms to survive: (1) mechanical persistence; (2) metabolic changes such as changes from an aerobic to an anaerobic state; (3) exchanging genetic information, for example, resistance genes; and (4) hiding from the immune system.

Multiple properties of *Staphylococcus epidermidis* and *Staphylococcus aureus* are involved in biofilm formation [9, 19]. The presence of extracellular DNA (eDNA), which is actively released through designated cells, has generated increased interest [25, 26]. The production of eDNA regulates the properties of extrapolymeric substances in response to environmental influences. Biofilm formation and production of extrapolymeric substances is related to an active release of eDNA, originating from bacteria that survived in an unfavorable environment. *S aureus* and *S epidermidis* rely on different autolytic mechanisms in which they use eDNA: eDNA produced by *S epidermidis* plays an important role during the attachment phase [21, 22]. This eDNA production is mediated by the autolysin protein AtIE that induces lysis of a small bacterial fraction enabling eDNA to be set free by *S epidermidis* during the surface attachment [4, 13]. In contrast, eDNA of *S aureus* originates from altruistic cell lysis and seems to be responsible for biofilm maturation, which is controlled by the *cid* operon [31]. Moreover, *S aureus* eDNA seems to establish a functional net structure in the biofilm matrix to tether cells together [6, 28]. Although the role of eDNA has been investigated in basic biofilm research [18, 22, 25], unresolved issues remain regarding eDNA in clinical biofilms: a remarkable variability of eDNA was reported in a study on clinical isolates of *S epidermidis* from patients with orthopaedic wound and implant infections [28].

This topic might be important to clinicians because prosthetic joint infection attributable to *S aureus* and *S epidermidis* are associated with frequent failure of long-term antibiotic treatment, revision surgery, and a debilitating course for patients. Determination of eDNA of the causative pathogen may be a new tool to identify bacterial strains with high eDNA content in biofilms as a potential therapeutic target or to adjust the treatment decisions, for example, early surgical intervention, to fight these infections more efficiently.

In the current study, we investigated the presence of eDNA in *S aureus* and *S epidermidis,* isolated either from patients with prosthetic joint infections or from control subjects without active infections. Using separate methods to observe DNA and eDNA and quantify eDNA and confocal laser scanning microscopy for observation, we asked: (1) Does DNA structure change during biofilm formation? (2) Are there time-dependent differences in eDNA production during biofilm formation? (3) Is there differential eDNA production between clinical and control Staphylococcal isolates? (4) Is eDNA production correlated to biofilm thickness?

## Materials and Methods

### Isolates and Strains Used in the Experiments

During 2000 to 2015, isolates from patients with infections of orthopaedic prostheses and implants were collected and stored at −80 °C. The explanted prostheses had to be transported from the operating theater to the microbiology laboratory under standardized conditions within no more than 2 hours to be included in our study. The explanted prostheses were placed in sterile boxes filled with phosphate buffered saline (PBS) and vortexed first for 30 seconds. Thereafter, boxes with implants were sonicated for 5 minutes and again vortexed for 30 seconds. The sonicated fluid was distributed on blood agar plates and cultured. Growing bacteria were counted and identified using routine microbiologic laboratory methods. Additionally the results obtained after implant sonication were confirmed with microbiologic cultures of intraoperative fluid samples to exclude possible contamination [14].

The pathogens of 60 patients (27 male, 33 female) were included in the study. The median age of the patients was 71 years (17–89 years). Thirty-one patients (51.5%) had infection of a primary prosthesis and 29 patients (48.5%) had infection of a secondary prosthesis.

Thus, overall 30 clinical isolates from infected hip prostheses (hip) (15 *S aureus* and 15 *S epidermidis*) and 30 isolates from infected knee prostheses (knee) (15 *S aureus* and 15 *S epidermidis*) were investigated. Control *S aureus* (n =15) and *S epidermidis* (n =15) isolates were collected from 30 volunteers without active infections who had not been exposed to antibiotics for 3 months and to a hospital environment during the previous 12 months. All *S epidermidis* isolates were collected from skin of the antecubital fossa. Control *S aureus* was isolated from the nares. All isolates were identified using standard microbiologic methods. *S epidermidis* DSM 3269 and *S aureus* American Type Culture Collection (ATCC^®^) 25921 (ATCC, Manassas, VA, USA; www.atcc.org) were used as reference strains.

### Characteristics of Clinical and Control Isolates

#### Susceptibility Testing

All bacteria were tested for susceptibility to oxacillin, rifampicin, ciprofloxacin, and vancomycin (disc diffusions test), using standard laboratory methods according to the European Committee on Antimicrobial Susceptibility Testing (EUCAST) criteria (http://www.eucast.org/clinical_breakpoints/).

### Biofilm Experiments

#### Biofilm Formation Testing

All experiments leading to biofilm formation were done on overnight cultures growing at 37 °C on Columbia agar + 5% sheep blood plates (bioMerieux SA, Marcy l’Etoile, France). After incubation the isolates and the reference strains were used for biofilm preparation using a modified protocol described by Christensen et al. [3]. Other than in the above-mentioned protocol, Brain Heart Infusion Broth (Sigma-Aldrich, St Louis, USA) was used for all experiments, as established in previous biofilm experiments [11, 12]. The 96-well flat bottom plates (Corning^®^ Costar^®^; Corning Life Sciences, Tewksbury, MA, USA) with bacterial suspensions were incubated for 24 hours at 37 °C. Biofilm quantification was determined by measuring the optical density after staining with crystal violet and counting the viable bacteria (viable bacteria count) in the biofilms.

For crystal violet staining, biofilms were fixed using 150 µL 2% glutaraldehyde/PBS. This fixing solution was chosen for its superior preserving qualities. The extinction of retained crystal violet was measured at 620 nm wavelengths using the FLUOstar^®^ Omega microtiter-plate reader (BMG LABTECH, Ortenberg, Germany). All biofilm experiments were done five times for each isolate to minimize variability. Additionally, confocal laser scanning microscopy was used to confirm biofilm formation.

To measure viable bacteria in biofilms, the supernatant containing planktonic cells was aspirated. The quantification of viable bacteria counts of *S epidermidis* and *S aureus* was assessed by serial dilutions; 100 µL of each dilution was plated onto blood agar plates. After 48 hours incubation at 37 °C, growth of colony forming units was counted.

### Detection of eDNA

Measurements of eDNA were performed on 24-hour biofilms using two methods: (1) fluorescence using 4′, 6-diamidino-2-phenylindole (DAPI), and (2) extraction of eDNA using the polymer mediated enrichment kit.

#### DAPI

DAPI is a fluorescence stain method to observe the DNA in cells. This staining method does not penetrate living bacterial cells such as staphylococci and was chosen to establish the amount of eDNA in living biofilms [7]. This procedure was performed overnight in black-bottom flat-well plates (Corning® Costar®; Corning Life Sciences). Biofilms grown for 24 hours were washed twice with PBS, air dried, and then stained with DAPI dissolved in PBS: 2 drops DAPI in 1 mL PBS (NucBlue^®^ Fixed Cell Ready Probes^®^ Reagent; Thermo Fisher Scientific, Waltham, MA, USA). To avoid cell penetration and thus staining intracellular DNA, staining was applied for 5 minutes only. The fluorescence of free eDNA in biofilms was measured immediately using the FLUOstar^®^ Omega microplate reader at 355/460 nm.

#### Extraction Using the Polymer Mediated Enrichment Kit

In this method 24-hour biofilms of the same isolate were scraped and pooled in 200 µL PBS. After vortexing for 20 seconds to break up the biofilms, the suspension was filtered using 0.22 Millex^®^-GS (Merck Millipore Ltd, Tullagreen, Ireland) filters in a glass tube. eDNA was extracted using the commercially available Polymer Mediated Enrichment Free-Circulating DNA Extraction Kit (Analytik Jena AG, Jena, Germany) according to the manufacturer’s instructions. The amount of extracted eDNA was measured using the NanoDrop^TM^ 2000c Spectrophotometer (Thermo Fisher Scientific).

### Confocal Laser Scanning Microscopy

#### Observation of 24-hour Biofilm Development in µ-Dishes in Dead Bacteria (reference strains)

To assess the role of eDNA during 24-hour biofilm development, 1 mL of each reference (diluted in Brain Heart Infusion Medium 1.5 × 10^7^), was cultivated in single 24-well Ibidi µ-Dishes (Ibidi Treat 1, 5 polymer coverslip, tissue culture treated; Ibidi GmbH, Planegg/Martinsried, Germany). Biofilms were grown at 37 °C for 24 hours on an orbital shaker. Every hour one well was taken off for further investigation. Biofilms were washed two times in PBS and fixed with 4% glutaraldehyde. First we observed general DNA production and its structure at different times of biofilm formation. Therefore, propidium iodide was used to observe the dense DNA of the dead bacteria (Molecular Probes^®^; Thermo Fisher Scientific). Polysaccharides, representing the most characteristic fraction of the extrapolymeric substances, were stained using concanavalin-A (ConA) (Sigma-Aldrich Corp, St Louis, MO, USA).

#### eDNA Observation on 6- and 24-hour Biofilms

To observe eDNA expression and its distribution in live biofilms, TOTO^®^-1 and SYTO^®^ 60 (Molecular Probes^®^) were used as recommended [24]. The working concentration of TOTO^®^-1 was 2 µmol/L and the counterstain SYTO^®^ 60 was used at a concentration of 10 µmol/L. TOTO^®^-1 stains the free eDNA surrounding living and dead cells, whereas SYTO^®^ 60 is able to penetrate only the cell wall of dead cells and stains the contained DNA red. Biofilms were grown in Ibidi 96-well µ-plates for optical microscopy. Confocal laser scanning microscopy was done after 6 and 24 hours. We used the same settings and region of interest for every investigation. The images were made using the LSM 780 confocal microscope system (Zeiss, Oberkochen, Germany). ImageJ (https://imagej.nih.gov/ij/) was used to score the images of stained biofilms. Every measurement was made in triplicate.

### Statistical Methods

Descriptive statistics, IBM SPSS^®^ Version 23.0 (IBM Corp, Armonk, NY, USA) was used to analyze the data. The unpaired two-sided Student’s t-test was used to compare eDNA production of the two staphylococcal species depending on the antibiotic exposure (control and clinical). Spearman’s rank correlation was used to assess eDNA production and biofilm thickness. Probability values less than 0.05 were considered statistically significant.

## Results

**Fig. 1A–E:**
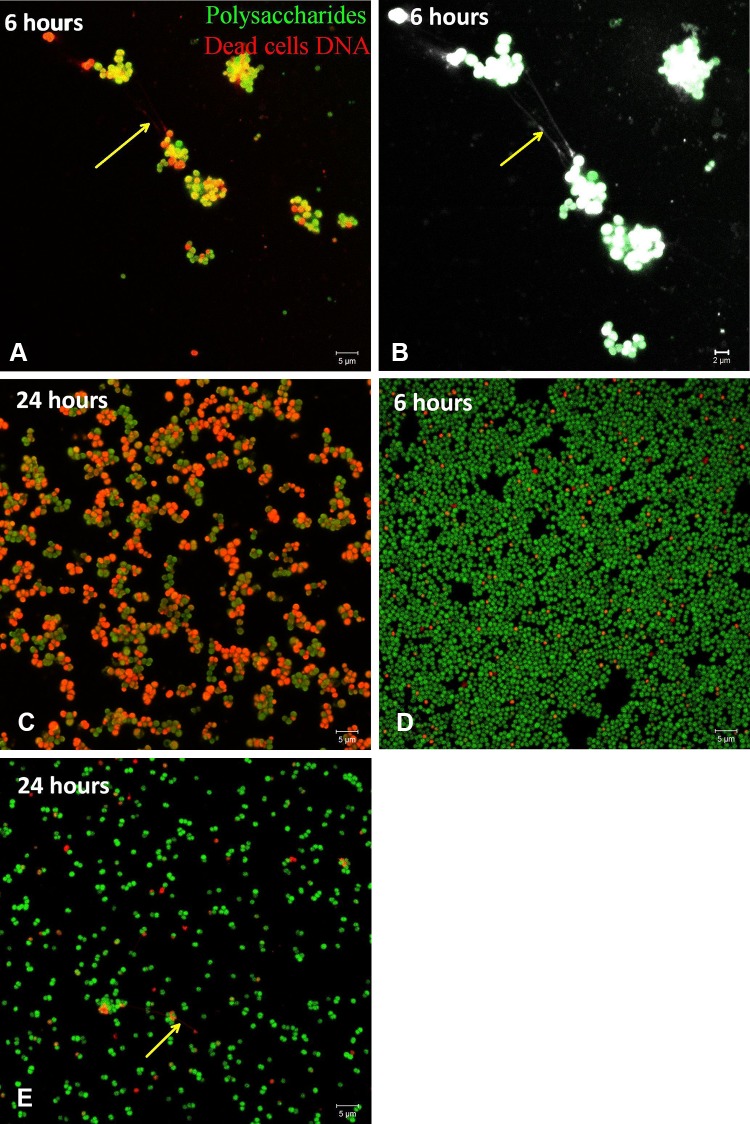
(**A**) The *S aureus* biofilm after 6 hours of incubation shows grape-like groups and suspected eDNA filaments (arrow) before spreading. (**B**) A magnification is shown of Illustration A, using a filter to better observe the filamentous bonds (arrow). (**C**) After 24 hours of incubation, a biofilm layer is forming. (**D**) The *S epidermidis* biofilm after 6 hours of incubation shows scattered cells over the surface. (**E**) After 24 hours of incubation, suspected eDNA filaments (arrow) are produced. (Original magnification, ×1000; scale bar: 5 µm).

During the 24-hour observation of the reference strains we observed differences in the pattern of biofilm formation: *S aureus* ATCC25923 aggregated and formed various grape-like aggregations of bacterial cells coated by single polysaccharides before spreading on the surface and forming a biofilm layer (Fig. 1A–B). In contrast, *S epidermidis* biofilms started with scattered cells spreading over the surface until reaching confluence and their maximum thickness at 24 hours without forming grapes or clusters (Fig. 1C–D). Most interestingly, we observed tiny filamentous bond-like structures between dead cells at different stages depending on the staphylococcal species. In *S aureus* biofilms the filaments were detected mainly after 5 to 6 hours of biofilm formation (Fig. 1A–B, arrow), whereas the filaments in *S epidermidis* biofilms were detected after 24 hours of biofilm formation (Fig. 1E, arrow).

eDNA staining of 24-hour biofilms with TOTO^®^-1 revealed less eDNA in biofilms of all *S aureus* isolates than eDNA in biofilms of all *S epidermidis* isolates (*S aureus* mean ± SD: 1.35% ± 2.0%; *S epidermidis* mean ± SD: 6.42% ± 10.6%; p < 0.05). The same staining showed greater production of eDNA in all clinical isolates at 6 hours regardless of their species (clinical isolates mean ± SD: 1.84% ± 1.31%; control mean ± SD: 1.17% ± 1.37%; p < 0.005). However, only biofilms of *S aureus* isolates showed differences in eDNA production at 6 and 24 hours (6-hour biofilms: clinical isolates mean ± SD: 1. 97% ± 1.51%; control mean ± SD: 0.88% ± 0.72%; p < 0.005; 24-hour biofilms: clinical isolates mean ± SD: 1.83% ± 2.30%; control mean ± SD: 0.38% ± 0.19%; p < 0.05). The amount of eDNA decreased in clinical and control biofilms of *S aureus* at 24 hours.

**Fig. 2A–D:**
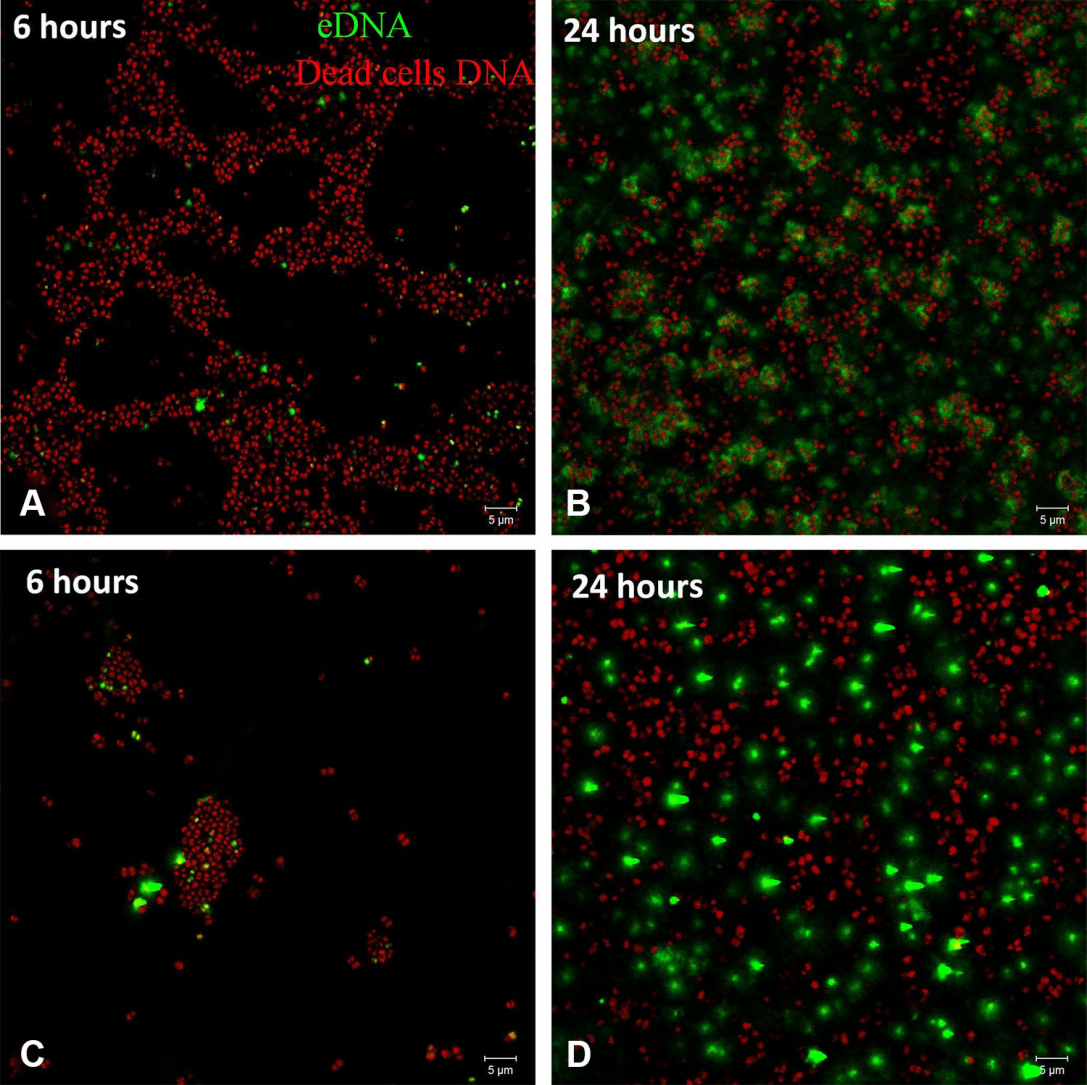
Clinical *S epidermidis* biofilms after (**A**) 6 hours and (**B**) 24 hours, and control isolates of *S epidermidis* biofilms after (**C**) 6 hours (**D**) and 24 hours are shown. The amount of dead cells DNA (red) measured after 6 hours was higher (p < 0.005) on biofilms of clinical isolates of *S epidermidis* than for control isolates. Generally eDNA (green) production was higher (p < 0.05) on all biofilms of clinical isolates than on control isolates. (Original magnification, ×1000; scale bar: 5 µm).

For *S epidermidis* there was no difference of eDNA between clinical isolates and controls (6-hour clinical isolates mean ± SD: 1.71% ± 1.07%; control mean ± SD: 1.45% ± 1.78%; 24-hour clinical isolates mean ± SD: 6.98% ± 12.62%; control mean ± SD: 5.3% ± 4.57%; (Fig. 2). The amount of eDNA at 24-hours was increased in clinical and control biofilms compared with 6-hour biofilms. A dense net of filamentous structures stained green with TOTO^®^-1 was seen on the confocal laser scanning microscopy image of *S epidermidis* on the 24-hour biofilms.

Regarding polymer mediated enrichment extraction on biofilms, all clinical isolates showed greater amounts of eDNA than biofilms of control isolates (clinical isolates mean ± SD: 88.6 ± 14.8 ng/µL versus control isolates mean ± SD: 69.6 ± 20.6 ng/µL; p < 0.005). In contrast, there was no difference in eDNA production between clinical and control biofilms using the fluorescence DAPI stain (clinical isolates mean ± SD: 2551 ± 909 fluorescence units; control mean ± SD: 2500 ± 1419 fluorescence units) (Table 1). Comparing clinical and control isolates of *S aureus* and *S epidermidis*, both methods showed concordant results: the amount of eDNA in biofilms of *S aureus* clinical isolates was greater than on biofilms of *S aureus* control isolates (p < 0.001) (Table 1). However, DAPI but not polymer mediated enrichment showed that eDNA in biofilms of *S epidermidis* clinical isolates was lower than on biofilms of *S epidermidis* control isolates (mean ± SD, 3292 ± 1520 fluorescence units; p < 0.05) (Table 1).

**Table 1:** Amounts of eDNA measured for clinical and control *S aureus* and *S epidermidis*

Type of isolate	DAPI (fluorescence units)	PME (ng/µL)	Confocal laser scanning microscopy mean % area eDNA (TOTO^®^-1; green)*	
			6-hour	24-hour
*S aureus* (n = 45)	2446 ± 2577	74.9 ± 21.6	1.61 ± 1.39	1.35 ± 2.00
*S epidermidis* (n = 45)	2622 ± 1113	89.6 ± 12.7	1.62 ± 1.33	6.42 ± 10.60
	No difference	No difference	No difference	p = 0.01
*S aureus*				
Clinical isolates (n = 30)	2814 ± 1058	84 ± 18.12	1.97 ± 1.51	1.83 ± 2.30
Control isolates (n = 15)	1707 ± 720	57 ± 16.2	0.88 ± 0.72	0.38 ± 0.19
	p = 0.001	p = 0.001	p = 0.002	p = 0.01
*S epidermidis*				
Clinical isolates (n = 30)	2287 ± 647	93 ± 8.5	1.71 ± 1.07	6.98 ± 12.62
Control isolates (n = 15)	3292 ± 1520	83 ± 16.4	1.45 ± 1.78	5.3 ± 4.57
	p = 0.005	p = 0.005	No difference	No difference
Clinical isolates (n = 60)	2551 ± 909	88.6 ± 14.8	1.84 ± 1.31	4.41 ± 9.36
Control isolates (n = 30)	2500 ± 1419	69.6 ± 20.6	1.17 ± 1.37	2.84 ± 4.04
	No difference	p = 0.001	p = 0.03	No difference

*Cyanine dimer TOTO^®^-1 to stain extracellular DNA (green); DAPI = 4’,6-diamidino-2-phenylindole; PME = polymer mediated enrichment.

Biofilm thickness determined using optical density depended mainly on the species, and whether the isolate was a clinical isolate or a control isolate from the skin of healthy control subjects. There were no differences between optical densities of biofilms of *S epidermidis* (0.42 ± 0.29) and *S aureus* (0.30 ± 0.19). Furthermore no differences between optical densities of biofilms of all clinical (0.40 ± 0.28) compared with control isolates (0.29 ± 0.18) were observed. The biofilm optical densities of *S aureus* clinical isolates (0.35 ± 0.2) were greater than those of *S aureus* control isolates (0.2 ± 0.13; p < 0.005). The biofilm optical densities of *S epidermidis* control isolates (0.37 ± 0.17) were greater than those of *S aureus* control isolates (0.2 ± 0.13; p < 0.005). Spearman’s rank correlation showed no relationship between production of eDNA by *S aureus* (clinical and control isolates) and biofilm production (rho = 0.29; p = 0.05). Similarly, no relationship was observed using Spearman’s rho between production of eDNA by *S epidermidis* (clinical and control isolates) and biofilm (rho = 0.12; p = 0.42).

Antibiotic susceptibility testing revealed less susceptibility to antimicrobials among the clinical isolates compared with controls. The clinical *S epidermidis* isolates were resistant to oxacillin (15/30), rifampicin (nine of 30), and ciprofloxacin (14/30). Control *S epidermidis* isolates were resistant to oxacillin and ciprofloxacin (three of 15). In comparison, clinical *S aureus* was resistant to oxacillin (eight of 30), ciprofloxacin (seven of 30), and rifampicin (one of 30). All tested isolates were sensitive to vancomycin (Table 2).

**Table 2:** Antimicrobial characteristics of the clinical and control isolates

Antibiotic resistance	Knee prosthesis n = 15	Hip prosthesis n = 15	Controls (nose) n = 15	Knee prosthesis n = 15	Hip prosthesis n = 15	Controls (cubital fossa) n =15
Oxacillin resistant	4 (27%)	4 (27%)	0	7 (47%)	8 (53%)	3 (20%)
Rifampicin resistant	0	1 (7%)	0	5 (33%)	4 (27%)	0
Ciprofloxacin resistant	2 (13%)	5 (33%)	0	7 (47%)	7 (47%)	3 (20%)
Vancomycin resistant	0	0	0	0	0	0

## Discussion

eDNA in biofilms plays an important role in biofilm formation and maturation [4, 18]. It is actively produced by a small bacterial population, and is essential for biofilm structure and stability [27]. In addition to its role in biofilm structure and stability, eDNA serves an additional role in antimicrobial resistance [7]. As such, eDNA may play a potential role in the diagnosis and treatment of biofilm-related infections. Using confocal laser scanning microscopy for observation, and separate methods to observe and quantify DNA and eDNA we asked (1) whether DNA structure changes during biofilm formation, (2) are there time-dependent differences in eDNA production during biofilm formation, (3) is there differential eDNA production between clinical and control Staphylococcal isolates, and (4) is eDNA production correlated to biofilm thickness?

One limitation of our study is that the observation method using confocal microscopy and the extraction method cannot be directly compared although both methods were used for different purposes. The extraction method is robust and may be used for experiments in future clinical studies. Additionally, the fluorescent stain SYTO^®^ 60 has a very short half-life making quantification problematic. The DAPI stain, which we intentionally used to selectively stain eDNA proved to be quite nonspecific in contrast to previous reports where DAPI penetrated the cells only after longer staining times [7, 16]. Finally, the use of brain heart infusion broth as the growth medium could be seen as a limitation since, in general, tryptic soy broth medium is used to enhance biofilm formation of *S epidermidis* producing polysaccharide intercellular adhesion [30]. Knowing that alternative pathways of biofilm formation based on the extracellular matrix binding protein (Embp) or through the release of eDNA are present, we opted for brain heart infusion broth based on our previous experiences with this medium [11, 12].

Propidium iodide stains DNA either in dead cells or dense eDNA structures [24]. Overall, eDNA is pivotal for the formation of biofilms in clinical isolates of *S epidermidis* and *S aureus* [22]. We observed filamentous structures stained by propidium iodide in *S aureus* (Fig. 1A, arrow) and *S epidermidis*, (Fig. 1D, arrow). Similar structures were observed in live biofilms of *S epidermidis* stained with TOTO^®^-1. TOTO^®^-1 stain is not cell permeable and exhibits a high DNA binding affinity owing to its four positive charges [24]. Furthermore, TOTO^®^-1 stain has no time constraints and it shows high sensitivity for observation of tiny and delicate eDNA structures. It may be possible that these filaments represent eDNA forming a network, as described by Dengler et al. [6].

TOTO^®^-1 staining to observe and quantify eDNA using confocal microscopy showed greater eDNA production in *S epidermidis* than in *S aureus* at 24 hours. An explanation for this finding may be that *S aureus*, being mostly a transient part of the human microbiota, starts biofilm formation only under certain conditions [23], whereas *S epidermidis* as a constant part of the human skin microbiota, relies on the constant production of eDNA to stabilize biofilms and to persist on different surfaces such as the human skin [10]. At 6 hours we detected substantially higher amounts of eDNA in biofilms of clinical isolates of *S aureus* and *S epidermidis* compared with control isolates, but not at 24 hours owing to the higher variance. The high amount of eDNA at 6 hours confirms previous studies in which *S aureus* used eDNA in the initial attachment and accumulation phase [15, 20, 29] . Qin et al. [27] indirectly supported this finding, stating that DNase, as DNA cleaving enzyme, was able to disturb or negatively influence biofilms during the first 6 hours of biofilm formation.

In the current study, biofilm thickness of all isolates was assessed using optical density measurements after crystal violet staining. According to our results, biofilm thickness did not correlate with eDNA production, as reported by Doroshenko et al. [7]. Biofilms of clinical isolates showed no differences in optical densities compared with biofilms of control isolates. Confocal laser scanning microscopy was most useful showing the growth pattern during biofilm formation.

Information regarding eDNA production of clinical isolates is scarce and our results indicate that clinical isolates of *S aureus* und *S epidermidis* produced substantially more eDNA than control isolates. Staining with TOTO^®^-1 and SYTO^®^ 60 allowed observation and quantification of eDNA using confocal laser scanning microscopy. Further research is needed to determine whether the amount of eDNA produced by clinical isolates might be considered as an additional diagnostic tool in staphylococcal biofilm infections. eDNA also might be a future target to modify or disrupt biofilms. Finally, characterizing pathogens by their eDNA production may serve as an outcome predictor of prosthetic joint infections and thus play a role in treatment decisions.
